# Systematic Review of the Management of Acute Type A Aortic Dissection with Mesenteric Malperfusion

**DOI:** 10.31083/j.rcm2405127

**Published:** 2023-04-24

**Authors:** Changtian Wang, Haiwei Wu, Zhilong Xi, Qiang Liu, Lei Sun, Lei Zhang

**Affiliations:** ^1^Department of Cardiovascular Surgery, Jinling Hospital, Affiliated Hospital of Medical School, Nanjing University, 210002 Nanjing, China

**Keywords:** acute type A aortic dissection, mesenteric malperfusion, surgical treatment, management strategy, outcome

## Abstract

**Background:**

Surgical treatment strategy for acute type A aortic dissection (aTAAD) with mesenteric malperfusion (MMP) is quite challenging as it is often associated with poor patient outcomes, and optimal management strategies remain controversial.

**Methods:**

We conducted MEDLINE and EMBASE database searches up to December 31, 2021 for studies on aTAAD with MMP. Data on study design, patient demographics, patient management strategy, mortality, complications, and follow-up were extracted, analyzed, and investigated.

**Results:**

Our literature search identified 941 potentially relevant studies, of which 19 were deemed eligible for this study. A total of 352 patients, mean age: 58.4 ± 11.9 years, diagnosed with aTAAD complicated with MMP were included with an overall prevalence of 4%. Patients for which MMP was observed preoperatively were also included in this analysis. The overall in-hospital mortality amongst these patients was 43.5%, and bowel necrosis and/or multiorgan failure were the major causes of death. Four management strategies for first-line treatment were recognized and these included central aortic repair (191, 54.3%), reperfusion of superior mesenteric artery (SMA) (121, 34.3%), exclusively endo-intervention (11, 3.1%), and exclusively medical intervention (29, 8.2%). These various first-line strategies showed mortality rates of 40.3%, 33.9%, 72.7% and 93.1%, respectively. There was no significant difference in the mortality rate between central aortic repair and reperfusion of SMA as first-line therapies ($\chi^{2}$ = 1.302, *p* = 0.254). When compared with central aortic repair and reperfusion of SMA, exclusively medical care exhibited a significantly greater mortality rate (*p *< 0.01).

**Conclusions:**

aTAAD complicated with MMP is a rare complication that carries a high mortality rate. Central aortic repair and reperfusion of SMA as first-line treatment strategies appear to be associated with better outcomes compared with exclusively endo-intervention and medical care. Clinical decisions may have introduced biases as no differences were indicated in regards to the way patients were being prioritized for the central aortic repair versus reperfusion of SMA. In regards to variable clinical features and pathology of aTAAD complicated with MMP, an individualized approach is recommended.

## 1. Introduction

Acute type A aortic dissection (aTAAD) is the most severe of aortic disease conditions and is associated with high mortality and morbidity. This condition requires prompt surgical intervention to prevent death from aortic rupture. Organ malperfusion is a catastrophic complication of aTAAD, and presents a great challenge for both disease diagnosis and management. Mesenteric malperfusion (MMP) secondary to aTAAD is a rare complication as it occurs in <5% of patients with aTAAD, but is a devastating complication and is strongly predictive of very poor patient outcomes [1, 2, 3, 4, 5, 6, 7, 8]. Hirst *et al*. [9] documented that involvement of either the superior mesenteric artery or celiac axis by aortic dissection was observed in 10% of these patients at autopsy. The in-hospital mortality rate of patients with mesenteric malperfusion is almost three times as high as that seen in patients without this complication (63 vs. 24%) [2]. In the analysis of the International Registry of acute Aortic Dissection (IRAD) which included 464 patients with aTAAD, mesenteric ischemia was the second most common cause of death (13.9%), only behind aortic rupture or cardiac tamponade (41.6%) [1]. In a recent report using IRAD data, the authors included 1809 consecutive patients with aTAAD and MMP was diagnosed in 68 patients (3.8%). The mortality of medical care, endovascular treatment and combined open and endovascular treatment was 95.2%, 72.7% and 41.7%, respectively, in aTAAD patients with MMP [2].

Initial central repair of the ascending aorta in an attempt to restore adequate true lumenal flow can mitigate malperfusion syndromes and avoid aortic rupture. However, given the existence of distal re-entry tears, persistence of false lumen flow, and possibility of branch vessel involvement, the restoration of proximal true lumen inflow may not reliably improve poor distal malperfusion. In addition, cardiopulmonary bypass (CPB), with or without hypothermic circulatory arrest (HCA), is associated with significant attenuation of visceral blood flow and activation of inflammatory processes, and therefore it enhances ischemia or reperfusion injury. With the development of transcatheter techniques, many centers have adopted a strategy of endovascular repair followed by aortic, or simultaneous, repair to restore superior mesenteric artery (SMA) blood flow first in aTAAD patients with significant MMP [3, 4, 10]. Delayed central aortic repair provides patients an opportunity to recover from malperfusion syndrome, and improves outcomes in this setting. However, the risk of aortic rupture, or complicated acute aortic valve insufficiency are elevated. Currently, the optimal treatment strategy for patients with aTAAD complicated with MMP is still controversial with respect to both the technical mode of first-line intervention, specifically central aortic repair versus reperfusion of SMA, and the relative timing of these therapeutic strategies.

The objective of this study was to investigate the status of the clinical management of aTAAD with MMP, and assess current evidence regarding various treatment strategies for this severe condition with the goal of improving patient outcomes.

## 2. Materials and Methods

### 2.1 Ethical Considerations

This systematic review was exempt from ethics approval as we collected and synthesized data published from previous studies in which informed consent had been obtained by the study investigators.

### 2.2 Search Strategy 

This review was non-registration protocol. It was conducted and reported according to the Preferred Reporting Items for Systematic Reviews and Meta-Analyses statement [11]. We searched MEDLINE through the PubMed portal and the EMBASE databases up to December 2021. Medical Subject Headings (MeSH) and text words were used for the searches and were supplemented by scanning the bibliographies of recovered articles.

The search strategy included a combination of keywords and MeSH including “mesenteric malperfusion”, “visceral malperfusion”, “organ malperfusion”, AND “acute type A aortic dissection”, “DeBakey Type I dissection”, “acute aortic dissection”. Two co-authors (CW and HW) reviewed and selected relevant articles independently for inclusion in this study. Differences of opinion between authors regarding included articles were resolved by consensus discussion. The references of selected articles were also reviewed to identify other potential articles for inclusion in this study.

Published studies were included if sufficient data regarding the number of patients who presented with aTAAD complicated with MMP, management strategies, and outcomes was provided. Only patients which MMP observed preoperatively were included. When registries or institutions published duplicate studies with extended length of follow-up or larger study populations, the latest and most complete study was included in this study to limit duplicate data. Case reports, reviews, and comments were excluded as well as reports that could not extract data precisely. Language was limited to articles written in English.

### 2.3 Data Abstract, Definitions, and Statistical Analysis

Data abstracted included various study characteristics such as study period, publication year, and institute, patient characteristics such as patient numbers, age, gender, and symptoms, the interval from onset of symptoms to operation (OSTO), and the first-line disease management strategy such as central aortic repair, reperfusion of SMA, exclusively endo-intervention, and exclusively medical intervention. Data abstracted also included surgical details such as the procedure conducted, cardiopulmonary bypass and cross clamp time, as well as in-hospital mortality and causes of death, postoperative complications, and patient follow-up.

We defined initial central aortic repair as the central surgery in initial treatment. Potential additional procedures included exploratory laparotomy, revascularization of SMA such as bypass, plasty, or stenting, as well as aortic stenting, fenestration, or thoracic endovascular aortic repair (TEVAR). We defined reperfusion of SMA as a first-line therapy as endovascular intervention, such as TEVAR, fenestration, or stenting, as well as bypass were often followed by or contemporaneous with central aortic repair surgery. Exclusively endo-intervention was defined as aortic or SMA stenting or fenestration, in which any other central surgical procedure was not performed. Exclusively medical intervention was defined as the patient receiving only medical care without any surgical or endovascular intervention.

The Cochrane’s Collaboration Risk of Bias Tool was used to assess the risk of bias at the study level, and categorize each study as high-risk, low-risk, or unclear-risk of bias [12].

Continuous variables were expressed as the mean ± standard deviation and categorical variables as percentages. The overall comparison of different strategies was performed using Chi-Square Tests. The subsequent pairwise comparison using partitions was conducted using the Chi-Square method. Statistical analysis was performed using SPSS version 18.0 (IBM Corp., Chicago, IL, USA).

## 3. Results

**Fig. 1.**
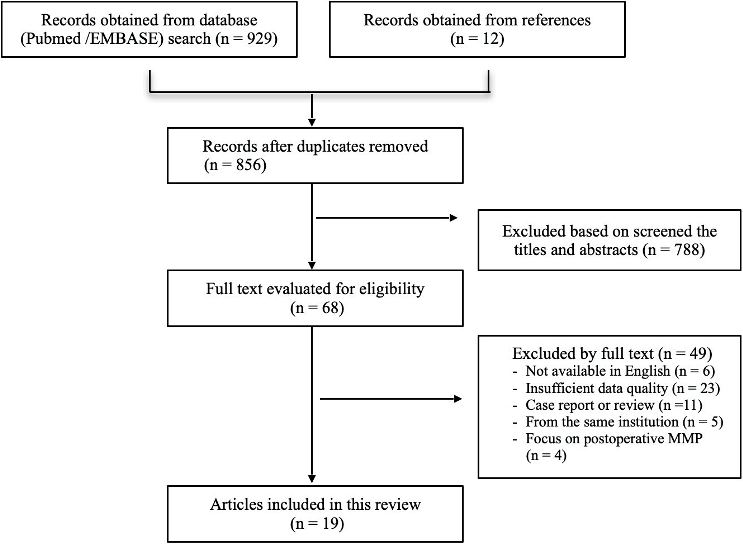
**Flow diagram illustrating the strategy used for identification, selection, and exclusion of articles used in this review**. MMP, Mesenteric malperfusion.

The literature search identified a total 929 studies (Fig. 1), and by manually retrieving the list of references, an additional 12 articles were identified. 68 papers were considered suitable for full text review after exclusion of duplicates or irrelevant studies. Nineteen studies were included in the final analysis [2, 3, 4, 5, 6, 7, 8, 10, 13, 14, 15, 16, 17, 18, 19, 20, 21, 22, 23], which were all retrospective studies spanning a period from 1963 to 2019 (Table 1, Ref. [2, 3, 4, 5, 6, 7, 8, 10, 13, 14, 15, 16, 17, 18, 19, 20, 21, 22, 23]).

**Table 1.** **The characteristics of studies on the management of aTAAD complicated with SMA malperfusion**.

First author	Study period	Published year	Institute	Study type	Total N. of patients	N. of SMA malperfusion patients (%)
Koizumi S [13]	2011–2019	2021	Kobe City Medical Center General Hospital, Japan	Case series	186	12 (6.4)
Okita Y [3]	1999–2017	2021	Kobe University, Japan	Case series	383	13 (3.4)
Yamasaki M [14]	2015–2017	2021	Tokyo CCU Network Scientific Committee, Japan	Case series	1504	14 (0.9)
Iannacone E [15]	1997–2019	2020	Weill Cornell Medicine, USA	Case series	336	7 (2.1)
Sugiyama K [16]	2017–2019	2020	Aichi Medical University Hospital, Japan	Case series	58	6 (10)
Yang B [10]	1996–2017	2019	University of Michigan Hospital, USA	Case series	602	82 (13.6)
Leshnower BG [4]	2003–2017	2019	Emory University School of Medicine, USA	Case series	618	34 (5.5)
Kawahito K [17]	1990–2016	2019	Jichi Medical University School of Medicine, Japan	Case series	1026	37 (3.6)
Chiu P [18]	2005–2015	2018	Stanford Hospital, USA	Case series	305	7 (2.3)
Uchida K [19]	2006–2016	2018	Yokohama City University Medical Center, Japan	Case series	438	12 (2.7)
Yamashiro S [20]	2000–2014	2015	University of the Ryukyus, Japan	Case series	121	10 (8.2)
Pacinia D [21]	2000–2008	2013	The Emilia- Romagna Registry, Italy	Case series	502	12 (2.4)
Di Eusanio M [2]	1995–2010	2013	IRAD, 18 referral centers worldwide	Case series	1809	68 (3.8)
Girdauskas E [5]	1994–2008	2009	Heart Center Leipzig, Germany	Case series	276	8 (3)
Shiiya N [22]	1991–2005	2007	Hokkaido University Hospital, Japan	Case series	84	5 (6)
Geirsson A [23]	1993–2004	2007	University of Pennsylvania, USA	Case series	221	3 (1.4)
Yagdi T [6]	1994–2003	2006	Ege University Hospital, Turkey	Case series	118	9 (7.6)
Lauterbach SR [7]	1990–1999	2001	Massachusetts General Hospital	Case series	101	5 (5)
Farm JI [8]	1963–1987	1990	Stanford University Medical Center, USA	Case series	128	8 (6.3)
Total	1963–2019			19	8816	352 (4)

Notes: aTAAD, acute type A aortic dissection; SMA, superior mesenteric artery.

### 3.1 Patient Demographics

A total of 352 patients diagnosed with aTAAD complicated with MMP were included in this investigation. The incidence of MMP was 4% in aTAAD patients, and the prevalence ranged from 0.9% [14] to 13.6% [10]. The median age of patients was 58.4 ± 11.9 years, and ranged from 31 to 87 years. 159 patients were male and 70 were female, and the sex of 123 patients were not reported. The clinical defects of MMP were poorly reported across all the included studies, with abdominal pain and metabolic acidosis with elevated lactate being the two major documented symptoms (Table 2, Ref. [2, 3, 4, 5, 6, 7, 8, 10, 13, 14, 15, 16, 17, 18, 19, 20, 21, 22, 23]).

**Table 2.** **Case series summary of the management of aTAAD complicated with SMA malperfusion**.

First author	N of patients	Age (years)	Sex (F)	Interval OSTO (hours)	Management Strategy	CCT (min)	CPB (min)	In-hospital mortality (%)	Causes of death	Postoperative Complications	Follow-up (mo)
Koizumi S [13]	12	60 ± 9.5	6	NA	S1	NA	NA	2 (16.7%)	MOF (n = 1); rupture (n = 1)	resection of the bowel (n = 4), stroke (n = 2), acute renal injury (n = 2), re-sternotomy for bleeding (n = 1)	NA
Okita Y [3]	13	63.7 ± 10.3	NA	3.7 ± 41.3	S1 (n = 8), S2 (n = 5)	NA	NA	6 (46.2%) (4 in S1; 2 in S2)	Bowel necrosis (n = 6), MOF (n = 2)	resection of the bowel (n = 6)	Actuarial survival after surgery at 2 years was 33.3 ± 18.02%
Yamasaki M [14]	14	NA	NA	3.9	S1	NA	NA	2 (14.3)	NA	NA	NA
Iannacone E [15]	7	NA	NA	NA	S1	90 (75–115)	148 (131–172)	2 (28.6%)	gangrenous bowel (n = 1); arresting (n = 1)	NA	NA
Sugiyama K [16]	6	58 (46–72)	1	NA	S1 (n = 2), S2 (n = 4)	NA	NA	0	0	paralytic ileus (n = 2), tracheostomy (n = 2)	NA
Yang B [10]	82	59.5 (50–68)	22	<24	S2	156 (127–191)	222 (185–261)	32 (39%)	MOF (n = 22), rupture (n = 11)	Reoperation for bleeding (n = 5); Postoperative MI (n = 2); AF (n = 24); New-onset CVA (n = 3); New-onset paraplegia (n = 1); Pneumonia (n = 17); Tracheostomy (n = 14); dialysis (n = 1)	10-year survival, 41%
Leshnower BG [4]	34	53 ± 13	8	NA	S1 (n = 16), S2 (n = 13), S4 (n = 5)	90, 131, 144, 178	151, 208, 214, 263	19 (55.8%) (9 in S1, 6 in S2, 4 in S4)	bowel necrosis (n = 13), died before op. (n = 3), protamine reaction (n = 1), stroke (n = 1), unknown (n = 1)	renal failure (n = 16), bowel necrosis or acidosis (n = 14)	NA
Kawahito K [17]	37	NA	NA	<48	S1	NA	NA	9 (24.3)	NA	NA	NA
Chiu P [18]	7	NA	NA	NA	S1	NA	NA	2 (28.6)	NA	NA	NA
Uchida K [19]	12	NA	NA	NA	S1 (n = 5), S2 (n = 7)	NA	NA	2 (16.7) (in S1)	bowel necrosis	NA	NA
Yamashiro S [20]	10	56.1 ± 13.4	6	4.8 ± 1.0	S1 (n = 2), S2 (n = 8)	164.4 ± 27.1	219.2 ± 30.6	2 (20) (in S1)	MOF	NA	115.8 ± 62.7 months; survival rate of 100% at 5 years
Pacinia D [21]	12	NA	NA	NA	S1	99.0 ± 43.5	185.2 ± 91.0	8 (66.7)	NA	NA	survival rate of 10% at 5 years
Di Eusanio M [2]	68	61.8 ± 14.4	21	19.1 (8.8–65.0)	S1 (n = 36), S3 (n = 11), S4 (n = 21)	NA	NA	43 (63.2) (15 in S1, 8 in S3, 20 in S4)	visceral ischemia (n = 15), neurologic (n = 2), MOF (n = 5), cardiac (n = 2), tamponade (n = 2), not specified (n = 15)	Brain injury (n = 5), Spinal cord injury (n = 1), MI/ischemia (n = 4), Acute renal failure (n = 20), Limb ischemia (n = 5), Cardiac tamponade (n = 5)	NA
Girdauskas E [5]	8	NA	NA	NA	S1	NA	NA	6 (75)	visceral ischemia	NA	24 months
Shiiya N [22]	5	79, 42, 68, 55, 40	2	NA	S1	NA	NA	0	0	NA	NA
Geirsson A [23]	3	NA	NA	NA	S1	NA	NA	1 (33.3)	MOF	no major complications	NA
Yagdi T [6]	9	NA	NA	36–48	S1	NA	NA	9 (100)	bleeding, sepsis, or MOF	NA	no
Lauterbach SR [7]	5	59, 64, 87, 62, 48	1	NA	S2 (n = 2), S4 (n = 3)	NA	NA	4 (80) (1 in S2; 3 in S4)	Necrotic bowel or not specified	short-gut syndrome	NA
Farm JI [8]	8	NA	NA	NA	S1	NA	NA	4 (50)	metabolic acidosis, myocardial failure, aortic rupture, renal failure, respiratory insufficiency	NA	NA
Total	352	58.4 ± 11.9 (31–87)	M 159, F 70	39.9 ± 74.9	S1 = 191, S2 = 121, S3 = 11, S4 = 29			153 (43.5%)			

Notes: AF, atrial fibrillation; aTAAD, acute type A aortic dissection; CCT, cross-clamp time; CPB, cardiopulmonary bypass; CVA, cerebrovascular accident; NA, not available; OSTO, onset of symptoms to operation; MI, myocardial infarction; MOF, multiorgan failure; SMA, superior mesenteric artery; S1, Central aortic repair as first-line treatment; S2, Reperfusion of SMA as first-line treatment; S3, Exclusively endo-intervention; S4, Exclusively medical care.

Overall, in-hospital mortality was 43.5% (n = 153), and ranged from 0 [16, 22] to 100% [6]. Bowel necrosis (43/153, 28.1%) and multiorgan failure (31/153, 20.3%) were the principal causes of death. The remaining causes of death included aortic rupture (12/153, 7.8%), neurologic complications (4/153, 2.6%), cardiac complications (including cardiac tamponade and arrest) (5/153, 3.3%), patient died before surgery (3/153, 2%), protamine reaction (1/153, 0.7%), and not specified (15/153, 9.8%). 38 patients did not have a documented cause of death.

5 studies reported patient follow-up. One study included 13 patients where the 2-year survival after surgery was 33.3 ± 18% [3]. Two studies reported 5-year survival rates of 100% [20] and 10% [21]. One report included 82 patients and indicated a 10-year survival rate of 41% [10].

Postoperative complications were partially documented across the included studies. Complications included postoperative bowel necrosis that required resection of the bowel or acidosis (n = 24), acute renal injury (n = 39), postoperative atrial fibrillation (n = 24), and pneumonia (n = 17) were reported. Other comorbidities included stroke, re-sternotomy for bleeding, tamponade, postoperative myocardial infarction, new-onset paraplegia, and paralytic ileus. Eleven studies which included a total of 129 patients did not document postoperative complications.

### 3.2 Management Strategies, Procedures, and Outcomes

A variety of treatment strategies for aTAAD complicated with MMP were encountered during this investigation. The first-line management strategies were classified as one of four types; specifically, central aortic repair (191, 54.3%), reperfusion of SMA (121, 34.3%), exclusively endo-intervention (11, 3.1%) and exclusively medical intervention (29, 8.2%) (Table 3).

**Table 3.** **Management strategies and outcomes for aTAAD complicated with SMA malperfusion**.

Death	Strategy				Total
	Central repair-first	Reperfusion of SMA-first	Exclusively endo-intervention	Exclusively medical care	
Survival	114 (59.7%)	80 (66.1%)	3 (27.3%)	2 (6.9%)	199
Death	77 (40.3%)	41 (33.9%)	8 (72.7%)	27 (93.1%)	153
Total	191	121	11	29	352

Chi-Square Tests, $\chi^{2}$ = 38.203, *p* = 0.000, According to the level of α = 0.05, the difference was statistically significant, and the mortality rate of the four strategies in the treatment of aTAAD complicated with SMA malperfusion was different. Central aortic repair as first therapy vs. reperfusion of SMA as first therapy $\chi^{2}$ = 1.302, *p* = 0.254. Central aortic repair as first therapy vs. exclusively endo-intervention $\chi^{2}$ = 3.252, *p* = 0.071. Central aortic repair as first therapy vs. exclusively medical care $\chi^{2}$ = 28.148, *p* = 0.000. Reperfusion of SMA as first therapy vs. exclusively endo-intervention $\chi^{2}$ = 4.960, *p* = 0.026. Reperfusion of SMA as first therapy vs. exclusively medical care $\chi^{2}$ = 33.104, *p* = 0.000. Exclusively endo-intervention vs. exclusively medical care $\chi^{2}$ = 1.451, *p* = 0.228. Notes: aTAAD, acute type A aortic dissection; SMA, superior mesenteric artery.

In-hospital mortality was 40.3% (77 of 191) and 33.9% (41 of 121) in patients with first-line therapy of central aortic repair or reperfusion of SMA, respectively. This difference was found to be not statistically significant ($\chi^{2}$ = 1.302, *p* = 0.254). In patients who underwent exclusively endo-intervention or received exclusively medical care, in-hospital mortality was 72.7% (8 of 11) and 93.1% (27 of 29), respectively. Again, these differences were not statistically significant ($\chi^{2}$ = 1.451, *p* = 0.228). Compared with first-line therapies of central aortic repair first or reperfusion of SMA, exclusively medical care had a statistically significant greater mortality rate (*p *< 0.01).

The exact causes of death, postoperative complications and survival rates of each group were not extracted. The details of the central aortic repair procedure including choice of arterial cannulation, core temperature during hypothermic circulatory arrest (HCA), cerebral perfusion strategy, HCA time, cross-clamp time, and CPB time were missing in most of the included studies. From this investigation, the interval from onset of symptoms to operation was 39.9 ± 74.9 hours in 7 studies, but most studies did not report this value.

## 4. Discussion

aTAAD complicated with MMP remains clinically challenging, commonly has poor outcomes, and optimal management practices are controversial. In this systematic review of 352 cases, the prevalence of MMP in aTAAD patients was low at 4%, but the pooled in-hospital mortality rate was very high at 43.5%. Five studies reported mortality rates greater than 50%, such as the study by Yagdi *et al*. [6], which reported an in-hospital mortality rate of 100%. However, this was a small cohort of 9 patients, and the majority of the deaths were bleeding, sepsis, or multiple organ failure. In contrast, some of the included studies reported lower mortality rates, for example, two studies Sugiyama *et al*. [16] and Shiiya *et al*. [22] reported in-hospital mortality rates of 0%. Both studies were comprised of a smaller cohort of patients and included only 5 and 6 patients, respectively.

As the two major surgical strategies for treatment of aTAAD with MMP, the survival of patients receiving central aortic repair first versus reperfusion of SMA was notably higher compared with conservative medical treatment (59.7%, 66.1% vs. 6.9%). In retrospect this makes surgical treatment superior to conservative medical treatment options.

Open surgical repair of the ascending aorta is a life-saving operation and remains the standard of care for patients with aTAAD, but is associated with a high rate of mortality in patients with MMP [2, 24, 25]. A long central repair surgery, with the patient on cardiopulmonary bypass with circulatory arrest, may accelerate ongoing intestinal ischemia and result in poor outcomes. In this review, the in-hospital mortality of central aortic repair as first treatment strategy was 40.3%, and this was found to be greater than reperfusion of SMA as the first strategy (34.3%). However, there was no significant difference in patient survival between these two approaches. This result is similar to several previous studies [2, 3, 4, 19, 20] which reported the in-hospital mortality of the reperfusion first strategy was lower than that of traditional central aortic repair first strategies. Mesenteric malperfusion time plays a role in determining outcomes in aTAAD patients, and expeditious reperfusion of SMA was crucial for favorable outcome. As a minimally invasive approach, percutaneous endovascular technique can quickly restore adequate blood flow to the obstructed arteries. The reperfusion as first treatment strategy is being gradually adopted as an alternative treatment option for aTAAD with MMP. This approach provides borderline patients the opportunity to recover and improve both short and long-term survival, as well as preventing a futile open aortic repair in the presence of unsalvageable organ damage and failure [26].

The premise of the reperfusion as first therapeutic strategy is possible in hemodynamically stable patients. However, hemodynamic instability is an important predictive factor for post-operative mortality in open surgical repair of the ascending aorta [25, 27]. Undoubtedly, patients with evidence of aortic rupture or cardiac tamponade who undergo central aortic repair contribute to the mortality rate of the central aortic repair as first therapy strategy patients. Of note, delaying open aortic repair in the treatment of aTAAD is controversial, and risks aortic rupture. For example, in the study by Yang *et al*. [10], the investigators reported eleven patients (13% of the study cohort) died from aortic rupture before receiving aortic repair surgery.

The period of medical stabilization from reperfusion of SMA to proximal aortic surgery is critical in weighing the risk of aortic rupture and inflammatory responses associated with CPB and adjunctive HCA and several authors have advocated for a strategy of simultaneous surgery [28, 29, 30]. A hybrid operating room [31] might address the reperfusion of vessel ischemia as first therapy followed by contemporaneous central aorta repair. Hybrid operating room (Hybrid OR) represents the ideal environment for teamwork between cardiovascular surgeon, vascular interventionist, and cardiac anesthesiologist. This concept offers a multidisciplinary opportunity for enhanced treatment of aTAAD with MMP. At present, there are no randomized studies on central aortic repair as the first treatment approach versus reperfusion of SMA, thus, and an individualized approach is essential. The American Association for Thoracic Surgery expert consensus document recommends it is reasonable to delay proximal aortic repair until after definitive treatment of mesenteric malperfusion (IIa, B) [24].

In IRAD, patients with MMP were less likely to undergo surgical treatment and more likely to receive medical or endovascular therapy [2]. As a treatment strategy, exclusively endo-intervention had a mortality rate of 72.7% and we measured a statistically significant difference when compared with an intervention of reperfusion of SMA as first therapy followed by central aortic repair. Exclusively medical therapy was associated with a dismal mortality rate (93.1%). Our study leads us to conclude that conventional central aorta repair as a standard treatment of aTAAD is essential.

An accurate diagnosis of MMP in aTAAD remains challenging. Only 60% of patients present with abdominal pain and no laboratory study can definitively confirm the presence, or absence, of mesenteric malperfusion [2]. Approximately 20% of patients without mesenteric malperfusion exhibited pain confirming that abdominal pain is a nonspecific symptom of acute mesenteric ischemia and, consequently, the diagnosis of MMP is frequently made too late to save the bowel and the patient [2, 25]. In this investigation, the time from onset of symptoms to operation varies greatly among the studies, and most studies do not record this time, thus, the optimal cutoff time on surgical intervention remains unclear. Accurate and timely diagnosis of MMP and prompt surgical intervention to restore the reperfusion of SMA might improve the poor outcomes. Hence, clinical investigation of data linking the onset of symptoms to surgery and corresponding outcomes are needed.

There are several significant limitations to consider when interpreting the results described in this study. Being a retrospective systematic review on management of aTAAD complicated with MMP, the data has inherent deficiencies. The risk of publication bias appears inevitable due to small samples in most studies, as well as the absence of preoperative clinical data and loss of patient follow-up. This may represent a number of different factors, such as different patient population or selection, differing centers with varying operator experience, or the different strategies used across the included studies. There were also variations in diagnosis of mesenteric malperfusion at different centers. Given these limitations, a general conclusion based on a solid statistical analysis with adequate sample sizes is not presently possible.

## 5. Conclusions

The available literature on aTAAD with MMP shows that aTAAD with MMP is a rare complication carrying a higher in-hospital mortality. Central aortic repair and reperfusion of SMA as the first therapeutic approaches appear to be associated with better outcomes compared with exclusively endo-intervention or medical management. Clinical decisions may have introduced biases showing no differences on the way patients were being prioritized regarding central aortic repair and reperfusion of SMA as the first therapy. With respect to the variation in presentation and pathology, an individualized approach is recommended.

## Data Availability

Data underlying the systematic review are retrieved from published studies and hence already available in literature; no unpublished data were employed. However, the collected data underlying this article will be shared on reasonable request to the corresponding author.

## Abbreviations

aTAAD, Acute type A aortic dissection; MMP, Mesenteric malperfusion; SMA, Superior mesenteric artery; TEVAR, Thoracic endovascular aortic repair.

## Supplementary Material

Supplementary material associated with this article can be found, in the online version, at https://doi.org/10.31083/j.rcm2405127.
